# Expression of Concern: Susceptibility mapping and zoning of highway landslide disasters in China

**DOI:** 10.1371/journal.pone.0349075

**Published:** 2026-05-11

**Authors:** The PLOS One Editors

The *PLOS One* Editors issue this Expression of Concern because this article [1] was identified as one of a series of submissions for which we have concerns about the peer review process. Readers are advised to interpret the article [1] with caution.

The Editors have no evidence of any author involvement in the peer review concerns.

In addition, the *PLOS One* Editors have concerns about the article’s compliance with the PLOS Authorship policy.
